# Deep learning framework for vertebral heart size and cardiothoracic ratio estimation in dogs and cats using thoracic radiographs

**DOI:** 10.3389/fvets.2025.1612338

**Published:** 2025-09-19

**Authors:** Habtamu Tilahun Mekonnen, Núria Puig, Alejandro Elson, Elena López, Paula Martínez, Selene Campos, Francisco Hernández, Daniel Mayor, Jorge Quilis, Xavier Cufí, Jordi Freixenet, Arnau Oliver, Xavier Lladó, Robert Martí

**Affiliations:** ^1^Computer Vision and Robotics Research Institute (ViCOROB), University of Girona, Girona, Spain; ^2^Integral Clínica Veterinaria Cullera, Valencia, Spain; ^3^Hospital Veterinario Bluecare, Málaga, Spain; ^4^Substrate AI, Valencia, Spain; ^5^4D Médica, Valencia, Spain

**Keywords:** dogs, cats, cardiomegaly, thoracic radiography, vertebral heart scale, cardiothoracic ratio, computer aided diagnosis, deep learning

## Abstract

**Introduction:**

Heart disease is a major cause of mortality in aging dogs and cats, with cardiomegaly being the most frequent radiographic finding. While deep learning methods have shown potential in detecting and quantifying cardiomegaly, their integration into clinical veterinary practice remains limited due to challenges in interpretability and workflow alignment.

**Methods:**

We developed a deep learning framework for the automatic estimation of Vertebral Heart Size (VHS) and Cardiothoracic Ratio (CTR) from thoracic radiographs of dogs and cats. A diverse dataset collected from two veterinary institutions was used. Segmentation of cardiac and thoracic anatomical regions was performed using Mask R-CNN, followed by automatic measurement of VHS and CTR. Model performance was evaluated against expert radiologist annotations.

**Results:**

The proposed framework demonstrated strong agreement with manual evaluations. Pearson correlation coefficients reached 0.922 for VHS and 0.933 for CTR, with regression slopes close to unity and minimal intercepts. The method was validated on both lateral and ventrodorsal projections, confirming its versatility across common clinical views.

**Discussion/conclusion:**

This work introduces an automated, robust approach for cardiac size assessment in dogs and cats. By supporting objective and reproducible measurements of VHS and CTR, the framework has potential to aid in the early detection and monitoring of heart disease, particularly in veterinary settings with limited access to specialized radiology expertise.

## 1 Introduction

Heart disease is a significant cause of mortality in aging dogs and cats (1). Proactive measures, including early detection and appropriate treatment strategies, are crucial in enhancing the quality of life and extending the lifespan of affected pets. While echocardiography remains the gold standard for diagnosing heart disease, providing detailed insights into cardiac structure and function, plain radiographs are commonly utilized for initial screening due to their rapid image acquisition and cost-effectiveness. Cardiomegaly, characterized by an enlarged cardiac silhouette on thoracic radiographs, is a common indicator of heart disease. It can be assessed using biomarkers such as the Vertebral Heart Size (VHS), also known as the Buchanan Index (BI), and the Cardiothoracic Ratio (CTR). VHS is a number that normalizes heart size to body size using mid-thoracic vertebrae as units of measure (2). It is evaluated from lateral thoracic radiographs of dogs or cats by measuring the longest axis of the cardiac silhouette, which extends from the tracheal carina to the cardiac apex, and the short cardiac axis at the widest part of the cardiac silhouette, perpendicular to the long axis. These measurements are then transferred to the vertebrae, starting from the cranial edge of the fourth thoracic vertebral unit (T4 vertebra), to count the vertebral units encompassed by each measurement and sum them to determine the value. CTR, on the other hand, is the proportion between the widest horizontal measurement of the heart and the widest horizontal measurement of the chest cavity (thorax), observed from the inner surface of the ribs on a ventrodorsal radiograph (3). In clinical veterinary practice, where access to highly-trained radiologists is often limited, the interpretation of radiographs frequently falls to less experienced veterinarians. This further highlights the challenges of manually assessing biomarkers like VHS and CTR, which are not only time-consuming and labor-intensive but also prone to measurement errors and interobserver variability.

To address these limitations, computer-aided diagnosis (CAD) systems have recently emerged to assist veterinarians in evaluating cardiomegaly, offering automated and consistent support for more accurate assessments. Yoon et al. (4) applied convolutional neural networks (CNN) and bag-of-features (BOF) to distinguish normal cardiac silhouettes from cardiomegaly, with CNN achieving superior performance, exceeding 95% in accuracy, sensitivity, and specificity. Building upon the success of CNN-based approaches, Burti et al. (5) developed a similar method to classify radiographs of dogs as having normal cardiac silhouettes (No-VHS-Cardiomegaly) or enlarged cardiac silhouettes (VHS-Cardiomegaly). Using models such as Inception V3, Inception-ResNet V2, VGG-19, and ResNet-101, they reported an area under the curve (AUC) exceeding 90% for all models, further emphasizing the potential of CNNs in this domain. Zhang et al. (6) extended these efforts by using high-resolution networks (HRNets) to detect 16 key anatomical landmarks (12 on vertebrae and 4 on the heart) in canine X-rays, enabling automated VHS calculation. This approach achieved an average performance (AP) of 90.9%, highlighting its robustness in facilitating cardiomegaly assessments. Similarly, Boissady et al. (7) employed a 121-layer DenseNet with attention mechanisms to predict cardiac and vertebral landmarks on lateral radiographs of cats and dogs. Their method achieved excellent agreement in VHS measurements between AI and human observers, with an intraclass correlation coefficient (ICC) of 0.998. In addition to VHS-based methods, alternative approaches have also been explored. Jeong and Sung (8) proposed a novel strategy for quantifying canine heart size using the adjusted heart volume index (aHVI). Their method combined attention U-Net for segmentation and advanced measurement techniques, achieving an AUC of 0.83 in detecting left atrial and ventricular enlargement. More recently, Li and Zhang (9) introduced a regressive vision transformer model with an orthogonal layer to predict VHS scores and classify canine cardiomegaly, achieving a classification accuracy of 87.3%. Finally, Zhang et al. (10) used diffusion models to generate synthetic images annotated with vertebral heart score key points, thereby expanding the training dataset. In addition, they employed a pseudolabeling strategy to identify high-confidence predictions, which were then used to iteratively refine the synthetic dataset.

Deep learning approaches have also been introduced to assist with CTR assessments for diagnosing cardiomegaly in human chest X-rays (CXRs). These methods predominantly rely on lung and heart segmentation to calculate CTR. Li et al. (11) demonstrated the effectiveness of a 2D U-Net architecture, achieving an accuracy of 95.3%. Chaisangmongkon et al. (12) evaluated four U-Net variants: VGG-11 U-Net, VGG-16 U-Net, SegNet, and AlbuNet, with AlbuNet demonstrating the highest accuracy of 96.32%. Ajmera et al. (13) employed an Attention U-Net, obtaining an accuracy of 94.96%, while Chou et al. (14) implemented AlbuNet-34, a U-Net variant incorporating ResNet as an encoder, achieving an accuracy of 94.9%.

While previous studies have demonstrated the effectiveness of deep learning in cardiomegaly classification and VHS estimation in veterinary radiographs, several limitations remain. Despite its clinical significance in human radiology, no prior veterinary study has implemented automatic CTR estimation. Existing studies have focused solely on automatic VHS estimation, which is limited to lateromedial (LM) radiographs, whereas ventrodorsal (VD) views provide crucial complementary information for cardiac assessment. Additionally, most cardiomegaly detection approaches have primarily emphasized classification or landmark detection rather than direct anatomical measurements, reducing their interpretability in clinical practice. Furthermore, these approaches are constrained by limited data diversity, which may not fully capture variations in imaging techniques, patient positioning, or breed differences, thereby affecting model generalizability.

Given these gaps, the objective of this study is twofold: (1) to develop a fully automated deep learning algorithm for VHS estimation from LM radiographs and CTR estimation from VD radiographs of dogs and cats in a robust manner, and (2) to evaluate the agreement between the automated predictions and those of human readers, thereby assessing the algorithm's reliability and consistency in clinical settings. To the best of our knowledge, the proposed work is the first to implement automatic CTR estimation in radiographs of dogs and cats. The main contributions of this study are summarized as follows:

1. Providing dual estimation of VHS and CTR to enhance clinical utility for comprehensive cardiac assessment;
2. Utilizing a diverse dataset from multiple veterinary institutions, capturing a variety of imaging techniques and patient demographics;
3. Integrating precise anatomical segmentation and measurement processes, offering reliable and interpretable metrics that align with clinical practice.
4. Conducting independent observer validation to ensure robust assessment of the automated measurements.

Beyond architectural novelty, our contribution lies in the clinical relevance, robustness, and integration of deep learning into a practical veterinary workflow. By combining anatomical segmentation with precise measurement of VHS and CTR, our end-to-end approach directly addresses real clinical needs in veterinary cardiology. It enhances interpretability, aligns with standard clinical practice, and demonstrates strong agreement with expert manual assessments, key factors for fostering trust and adoption in clinical settings.

## 2 Materials and methods

### 2.1 Materials

#### 2.1.1 Database construction

Thoracic radiographs of dogs and cats were collected from routine clinical examinations conducted at two veterinary centers: Integral Clínica Veterinaria Cullera and Hospital Veterinario Bluecare. These examinations utilized Sedecal Neovet SHF-210 X-ray generation systems, equipped with the E7239 X model X-ray tube. The acquisition system comprised a Vieworks Vivix-S 4343V wired digital radiology detector paired with DxWorks (VxVue) acquisition software. Both the detector and the software are CE Mark-certified. For standardization, animal positioning during radiographic acquisition was guided by a Spanish manual (15). Radiographic techniques adhered to the ALARA principle (as low as reasonably achievable), minimizing radiation exposure while ensuring diagnostic quality.

#### 2.1.2 Training and validation datasets

This study used LM and VD thoracic radiographs of dogs and cats acquired between September 2023 and April 2024. Technicians at each center performed a preliminary quality check, and images were selected based on predefined quality criteria. Radiographs were excluded if they did not depict thoracic regions, showed abnormal or incomplete fields of view, exhibited incorrect positioning, or had poor exposure. Following this evaluation, 199 LM and 200 VD images were selected from the picture archiving and communication systems (PACS) of the institutions. These numbers reflect both the patient volume at the veterinary centers and the data volume necessary for training the deep learning algorithm. The dataset was then split into training and validation subsets with an 80–20 ratio.

Table 1 summarizes the dataset used. Notably, we intentionally do not differentiate between dogs and cats images. Given that both species undergo the same clinical imaging protocol and that the primary objective of this study is to develop a generalizable framework applicable across species, we combined the data into a single, more heterogeneous dataset.

**Table 1:** Summary of training and validation datasets used for VHS and CTR estimation.

**View postion**	**Training**	**Validation**	**Total**
LM	159	40	199
VD	160	40	200

#### 2.1.3 Test dataset and observer study dataset

The test dataset consisted of 144 radiographic images, including 39 LM and 37 VD images from Integral Clinica Veterinaria Cullera, and 37 LM and 31 VD images from Hospital Veterinario Bluecare. VHS for LM images and CTR for VD images were manually calculated by each center specialist team. In total, 5 veterinary specialists participated in the annotations, 3 from Bluecare and 2 from Cullera Clinic, with varying levels of expertise. Each team included an expert veterinary radiologist, supported by veterinary anesthesiologists and neurologists. To minimize inconsistencies in annotations, an additional validation step was performed by the expert radiologist at each center. Moreover, regular coordination meetings between teams helped to harmonize imaging quality protocols and improve annotation consistency. These manual assessments served as a reference for evaluating the performance of the proposed automated estimation method.

From this test set, we conducted a inter-observer study by having the specialists from one center evaluate images from the other. This observer study dataset comprised a total of 56 images, 20 VD images, and 5 LM images from Integral Clinica Veterinaria Cullera, and 19 VD images and 12 LM images from Hospital Veterinario Bluecare.

#### 2.1.4 Data preprocessing

The workflow began with the automatic extraction of key attributes from the DICOM tags, including filename, patient number, view position, and patient comments. Depending on the view position, specific ground-truth annotations were then recorded: for LM images, these included VHS, cardiac long and short axis lengths, and the number of vertebral units occupied by each axis starting from the T4 vertebra; for VD images, these included CTR and the transverse diameters of both the thorax and heart. To minimize transcription errors, DICOM metadata extraction was automatically performed and verified against a random sample, while all manual annotations were stored in a standardized CSV format. Finally, the CSV file was cross-checked by an expert veterinary radiologist. Regarding patient information and anonymization, only the patient number (numerical code) was retained to link cases across datasets while ensuring confidentiality.

**Figure 1:**
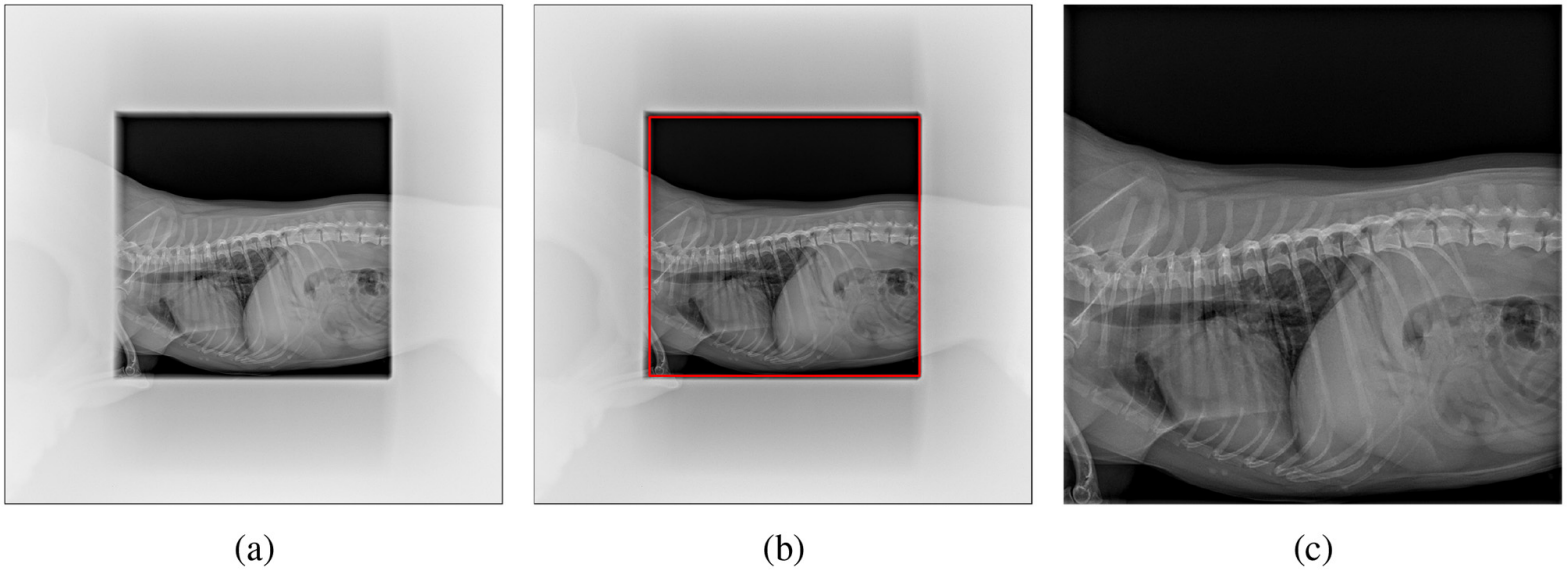
Cropping the pet's X-ray scan: **(a)** original image with non-relevant regions blurred for clarity, **(b)** region of interest (RoI) detected by YOLO highlighted in red, and **(c)** final cropped image used for analysis.

The radiographs, initially in DICOM format, were converted to Portable Network Graphics (PNG) format for further processing. This conversion took into account the photometric interpretation tag, with windowing applied as needed. Subsequently, the images underwent normalization, with their intensity values rescaled to 8-bits. To ensure that only relevant information is analyzed, the images were automatically cropped to focus on the region of interest (RoI), the central rectangular region containing the patient's thorax, excluding the surrounding brighter area, as depicted in Figure 1. You Only Look Once version 8-m (YOLOv8m), a convolutional network-based object detection model pre-trained on the COCO (Common Objects in Context) dataset, was employed to identify and locate the RoI. YOLOv8m was chosen among different variants of YOLOv8 to have a good balance of speed and accuracy. The model was trained using Ultralytics,^1^ a library providing an optimized implementation of YOLO, offering various tools and functionalities for object detection and deep learning workflows. The training was performed in batches of eight images using the stochastic gradient descent (SGD) optimizer. Ultimately, the bounding box predicted by the trained model was used to crop the image, isolating the thoracic region for focused analysis. This initial automatic crop was also subsequently validated and refined by clinicians.

#### 2.1.5 Data annotation

Manual annotations were performed to provide accurate ground-truth data for model training and validation, ensuring reliable segmentation of the relevant anatomical structures. Specifically, in LM images, the heart, the sixth thoracic vertebral unit (T6 vertebra), and the carina were annotated, while in VD images, the heart and thorax were annotated. Labelme,^2^ an open-source graphical annotation tool for image and video labeling that supports polygonal, rectangular, and keypoint annotations (16), was used for precise delineation of these anatomical structures. Polygon annotations were initially saved in JSON format and subsequently converted to COCO format to ensure compatibility with the training pipelines. These annotations were then used to train and validate segmentation models designed to detect anatomical regions essential for the estimation of VHS and CTR. All annotations were reviewed by two experienced radiologists to eliminate potential errors.

It is important to note that the test set did not include these segmentation annotations, but rather VHS and CTR values manually computed by the expert teams. Consequently, the evaluation of automatic VHS and CTR estimation was performed using the observer study dataset, which served as the benchmark for assessing the accuracy of our approach.

### 2.2 Methods

**Figure 2:**
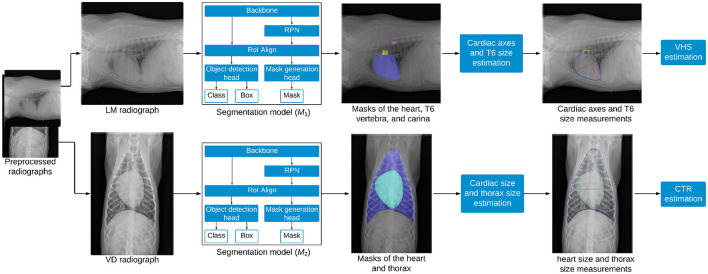
Overview of the methodology for estimating VHS and CTR from thoracic radiographs. LM images are processed by the segmentation model *M*_1_ to segment the heart, trachea, and T6 vertebra, enabling the estimation of VHS, while VD images are processed by the segmentation model *M*_2_ to segment the heart and thorax, facilitating the estimation of CTR.

The overall methodology employed in this study is depicted in Figure 2. It includes three key steps: segmentation of different regions in the thorax, estimation of anatomical features, and calculation of the biomarkers.

#### 2.2.1 Segmentation model

Mask R-CNN (17), a popular deep learning technique for object instance segmentation, was employed in this study to segment the heart, T6 vertebra, and carina from LM images, as well as the heart and thorax from VD images. The model is composed of several key components: a backbone, a Region Proposal Network (RPN), a Region of Interest Alignment (RoI Align) layer, an object detection head, and a mask generation head, as shown in Figure 2. The ResNet-50 backbone with a Feature Pyramid Network (FPN) architecture was adopted as a feature extractor. FPN is a network architecture that addresses scale variance in object detection and instance segmentation by combining low-level and high-level features from different stages of a backbone network to create multi-scale feature maps for detecting objects of various sizes. When a radiograph is fed into the ResNet backbone, it passes through multiple residual bottleneck blocks, resulting in a feature map that encapsulates abstract representations of the image. The RPN scans this feature map to propose regions likely to contain anatomical structures of interest, employing anchor boxes of varying scales and aspect ratios to densely cover spatial locations in the input image. These anchor boxes are then refined based on learned features, serving as reference bounding boxes for subsequent processing. The RoI Align layer then extracts feature vectors from the feature map based on the RoIs proposed by the RPN and transforms them into a fixed-size tensor. This refined feature map is processed by two parallel branches: the object detection branch and the mask generation branch. In the object detection branch, the model predicts the object category and refines the instance bounding box for each RoI using a fully connected layer that maps feature vectors to a final set of *n* object classes and 4*n* corresponding bounding box coordinates. Meanwhile, the mask generation branch operates as a fully convolutional network, where the RoI feature map is passed through a transposed convolutional layer and a convolutional layer to produce a binary segmentation mask for each class. For each anatomical structure of interest, the mask head outputs a probability map for each pixel within the bounding box region of the structure. These probability maps are subsequently thresholded using a value of 0.5 to create binary masks, precisely delineating the exact pixels occupied by each structure. Two distinct Mask R-CNN models were trained in this study: *M*_1_ for segmenting the heart, T6 vertebra, and carina from LM images, and *M*_2_ for segmenting the heart and thorax from VD images.

#### 2.2.2 VHS and CTR estimation

After obtaining the segmentation masks of the heart, T6 vertebra, and tracheal carina from *M*_1_, the contours of each region were identified to facilitate further analysis. The intersection region between the tracheal carina and the heart was determined using their respective contours. Within this intersection, the carina point was defined as the central point along the x-axis and the uppermost point along the y-axis. The apex point, identified as the farthest point on the heart contour from the carina point, was then located. The long axis (*L*) of the cardiac silhouette was measured as the distance between the carina point and the apex point, while the short axis (*S*) was measured at the widest part of the cardiac silhouette, perpendicular to the long axis. To estimate the size of the T6 vertebra (*T*_6_), the major axis of an ellipse fitted to the T6 vertebra contour was measured. Finally, VHS was calculated using Equation 1, where the sum of the cardiac long and short axes was divided by the size of the T6 vertebra. Only Jeong and Sung (8) has proposed the use of a single vertebra (specifically T4) as a normalizing factor for a novel cardiac index. Building on this approach and based on our initial experiments, we proposed using the T6 vertebra to normalize the patient's heart size. Our findings demonstrate that the T6 vertebra provides better results than the T4 vertebra (see Section 4), and to the best of our knowledge, this approach has not been previously proposed.

(1) $$\begin{array}{c} VHS=\frac{L+S}{T_{6}} \end{array}$$

**Figure 3:**
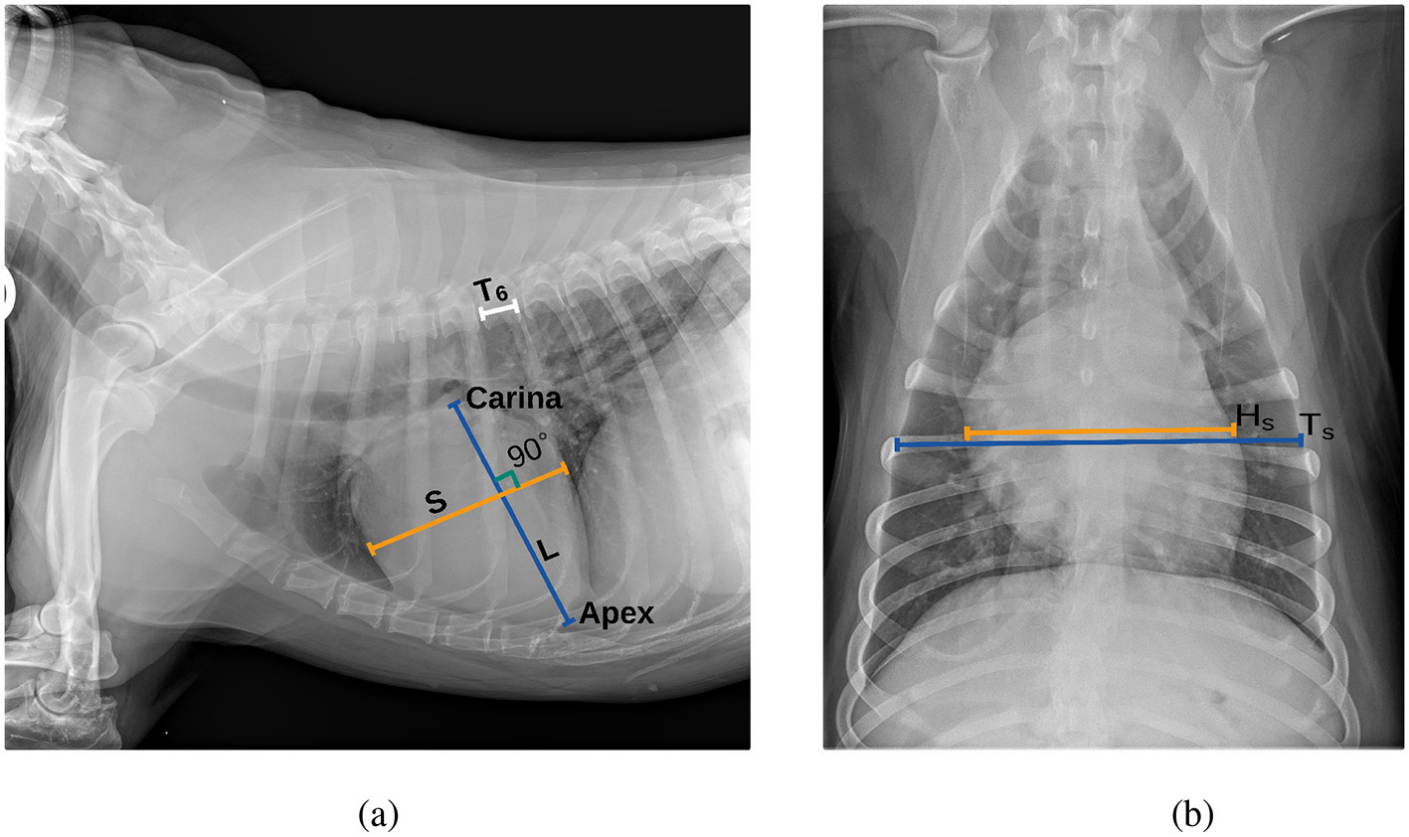
Thoracic radiographs illustrating cardiac assessment. **(a)** Lateral projection demonstrating the cardiac axes and T6 vertebra used for VHS estimation. **(b)** Ventrodorsal projection depicting heart and thorax dimensions for CTR estimation.

Once the segmentation masks of the heart and thorax were obtained from *M*_2_, the contours of both regions were identified and smoothed using B-spline interpolation to ensure accurate boundary representation. The widest transverse dimension of the heart (*H*_*s*_) was then measured as the distance between the leftmost and rightmost points on the heart contour. Similarly, the widest transverse dimension of the thorax (*T*_*s*_) was measured as the distance between the leftmost and rightmost points on the thorax contour. Finally, CTR was calculated as the ratio of the heart's widest transverse dimension to that of the thorax, as defined in Equation 2. The process of measuring the cardiac axes and the T6 vertebra for VHS estimation, as well as the heart and thorax dimensions for CTR estimation, is shown in Figure 3.

(2) $$\begin{array}{c} CTR=\frac{H_{s}}{T_{s}} \end{array}$$

#### 2.2.3 Evaluation metrics

For algorithm development and initial quantitative evaluation, we used the manual segmentations performed under expert supervision, providing precise anatomical references necessary for model training. However, the core of the clinical evaluation and the algorithm reliability assessment was performed using the annotations directly obtained from experienced veterinary radiologists. This expert-based evaluation is crucial, as it ensures that the algorithm's performance is measured against the highest clinical standards, thereby validating its practical utility and trustworthiness in real veterinary practice.

Therefore, on the one hand, the Dice Similarity Coefficient (DSC) was employed to evaluate the performance of the segmentation models on the validation set. The DSC quantifies the similarity between the predicted segmentation mask and the ground-truth mask on a pixel-by-pixel basis. It ranges from 0 to 1, where 0 indicates no overlap and 1 represents perfect overlap. Higher DSC values correspond to better segmentation accuracy and improved model performance. The DSC is computed according to Equation 3, where |*P*∩*G*| denotes the number of pixels common to both the predicted mask PP and the ground-truth mask *G*, while |*P*| and |*G*| denote the total number of pixels in the predicted and ground-truth masks, respectively:

(3) $$\begin{array}{c} DSC=\frac{2\;|P\cap G|}{\left|P|+|G\right|} \end{array}$$

On the other hand, to assess the clinical similarity between VHS and CTR measurements, two complementary metrics were used. First, the Root Mean Squared Error (RMSE), defined as the square root of the average squared differences between ground-truth and estimated values, quantifies the magnitude of deviations between automatic and manual VHS and CTR estimates. Second, the Pearson Correlation Coefficient (PCC) was calculated to evaluate the linear correlation and similarity between these measurements. In addition, we used Bland-Altman plots to analyze the inter-observer study.

#### 2.2.4 Experimental settings

The segmentation models were trained using Detectron2 (v0.6), an open-source computer vision library built on PyTorch. To accelerate training and enhance performance, transfer learning was employed by initializing the models with pre-trained weights from the COCO dataset. COCO is a widely recognized benchmark dataset for research in object detection, instance segmentation, and image captioning. It comprises 328,000 images of everyday objects and humans, annotated with bounding boxes and segmentation masks for 91 distinct object categories. Leveraging the pre-trained weights enabled the models to utilize generic low-level features, such as edges and textures, learned from a large dataset, while fine-tuning adapted these features to the specific requirements of our domain. This approach effectively transferred pre-trained knowledge to our domain-specific task, mitigating challenges associated with limited training data.

For this experiment, the shorter side of the input images was resized to 800 pixels, while the longer side was constrained to a maximum of 1,333 pixels, preserving the aspect ratio. These settings ensured that images were uniformly scaled, facilitating consistent training. Additionally, data augmentation was employed by randomly flipping images horizontally with a 50% probability, enhancing the model's robustness to variations in the data. The training was conducted on an NVIDIA A30 GPU with 24 GB of memory, utilizing CUDA for accelerated computation. The model was trained with a batch of 2 images and optimized using stochastic gradient descent (SGD) with a momentum of 0.9 and a weight decay of 0.0001. The loss function consisted of a combination of cross-entropy loss for classification, smooth L1 loss for bounding box regression, and binary cross-entropy loss for mask prediction, enabling comprehensive optimization across different components. Performance monitoring and early stopping were conducted using the mean Average Precision (mAP) calculated over a range of IoU thresholds from 0.5 to 0.95, with a step of 0.05. The training parameters used to finetune the segmentation models are described in Table 2.

**Table 2:** Summary of training parameters for models M1 and M2, including the learning rate, number of training epochs, and the number of sampled RoIs per image during the training process.

**Model**	**Learing rate**	**Epochs**	**Sampled RoIs per image**
*M*_1_	0.0025	40	512
*M*_2_	0.0025	56	256

## 3 Results

We first evaluate the performance of the deep learning models for VHS and CTR evaluation using the validation dataset. Subsequently, we evaluate the clinical significance of the algorithm using the independent test dataset. We finalize the evaluation comparing our algorithm with the observer study.

### 3.1 VHS estimation model

#### 3.1.1 Performance in segmentation of the heart, T6 vertebra, and carina

**Figure 4:**
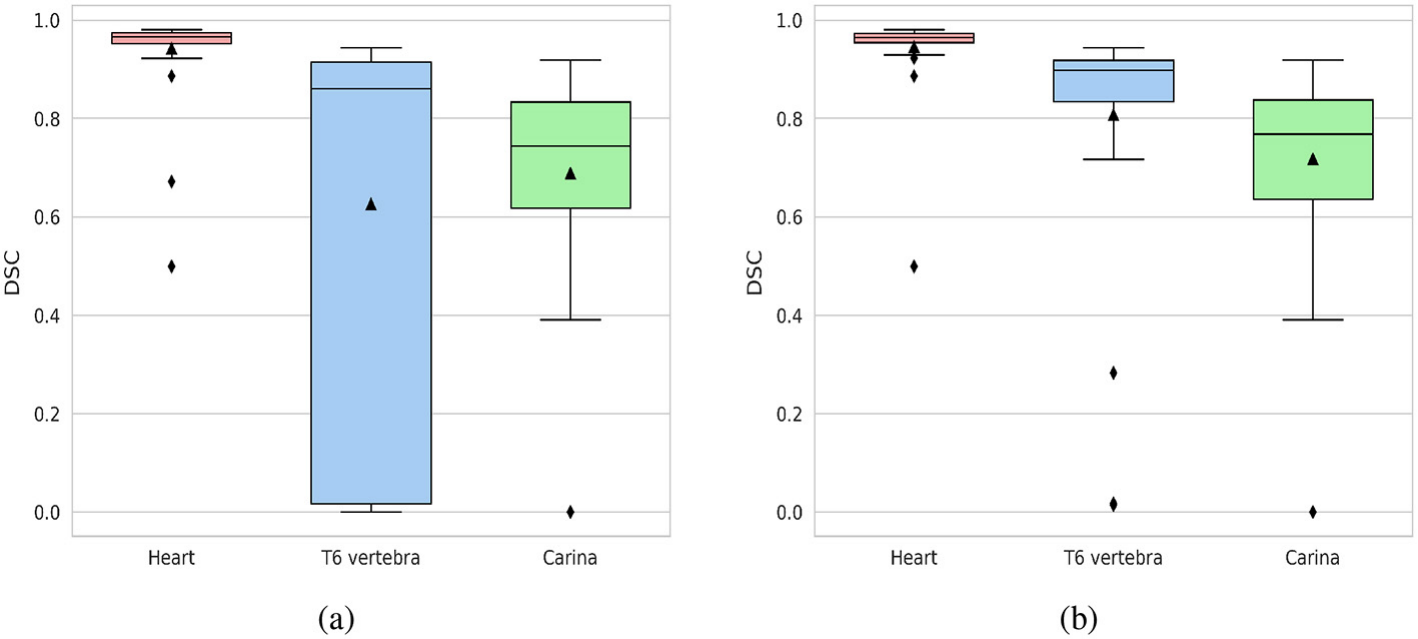
Box plots of DSC for the segmentation of the heart, T6 vertebra, and carina. **(a)** DSC distribution for the entire validation dataset (40 cases). **(b)** DSC distribution with cases of T6 vertebra mis-segmentation excluded (31 cases).

The mean DSC for the segmentation of the heart, T6 vertebra, and carina in the validation dataset was 94.2%, 62.6%, and 68.9%, respectively. The high DSC achieved for heart segmentation highlights the model's effectiveness in accurately capturing the anatomical features of the heart within the images. With a median dice score of 87%, the segmentation model performs well in capturing and delineating the structure of the T6 vertebra in most cases. However, in some cases, the model segmented the fifth thoracic vertebral unit (T5 vertebra) or seventh thoracic vertebral unit (T7 vertebra) instead of the T6 vertebra, leading to a lower mean DSC. This mis-segmentation arises due to the relative positioning and structural similarities among T5, T6, and T7 vertebrae. Despite this, the use of a specific vertebra, such as T6, is not critical for the normalization process in the VHS calculation, as the T5, T6, and T7 vertebrae exhibit similar dimensions. The normalization process is generally robust to minor positional changes or variations introduced by selecting an adjacent vertebra. By excluding 9 cases where the model mis-segmented the T5 or T7 vertebra instead of the T6 vertebra, the mean DSC for the T6 vertebra improves significantly from 62.6% to 80.8% as shown in Figure 4. Excluding the mis-segmented cases demonstrates the model's potential to achieve high segmentation performance for the T6 vertebra, emphasizing that the primary challenge lies in distinguishing adjacent vertebrae rather than the segmentation process itself. The dice score for carina detection is considered acceptable, as the focus is on achieving overall localization of the region.

#### 3.1.2 Performance of VHS calculation

**Figure 5:**
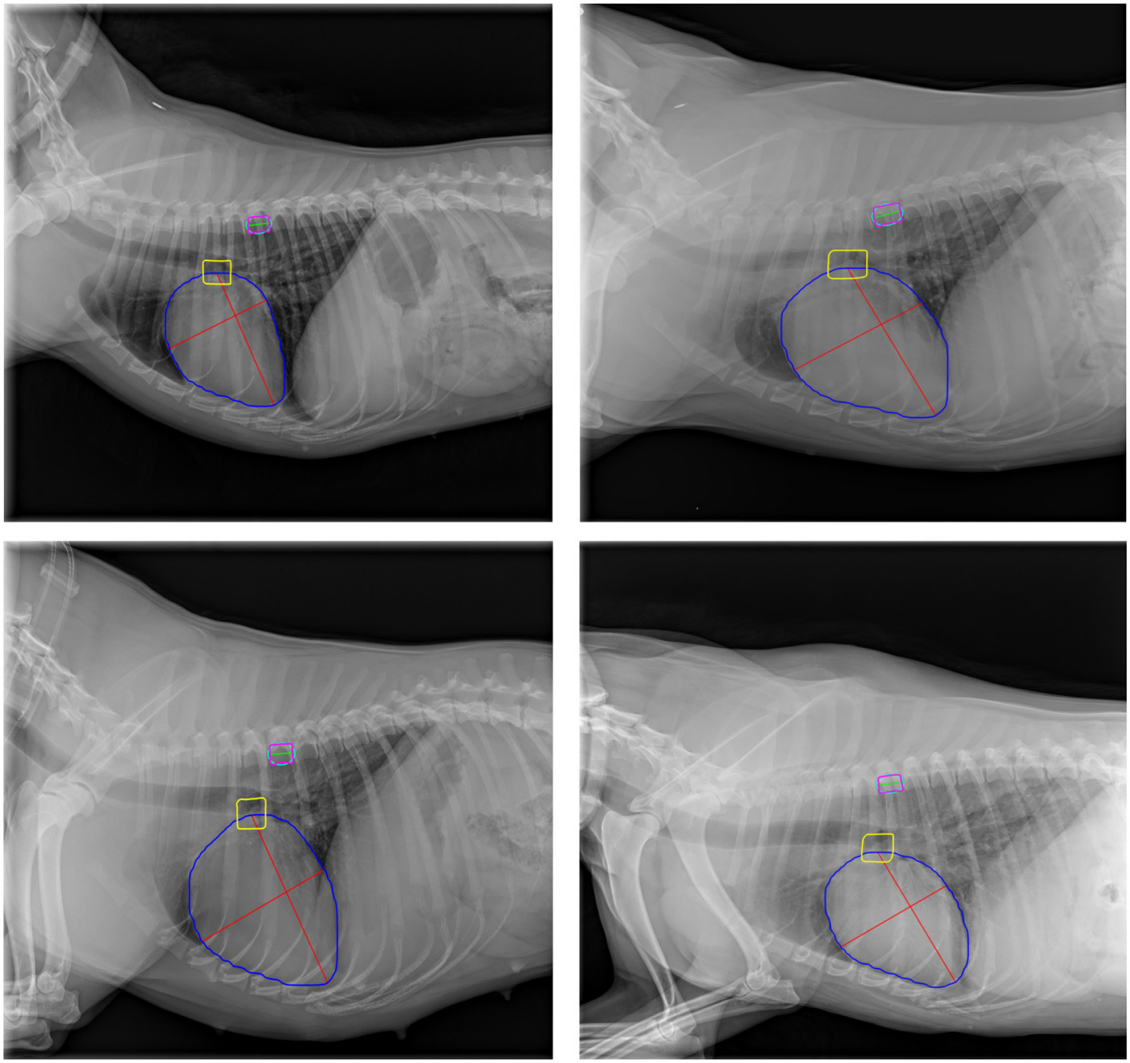
Representative examples of cardiac axes and T6 vertebra size estimation on validation images. Blue, yellow, and magenta contours represent the heart, tracheal carina, and T6 vertebra, respectively. The cyan outline depicts the ellipse fitted to the T6 vertebra contour. Red lines indicate the cardiac axes, while the green line represents the T6 vertebra size.

**Figure 6:**
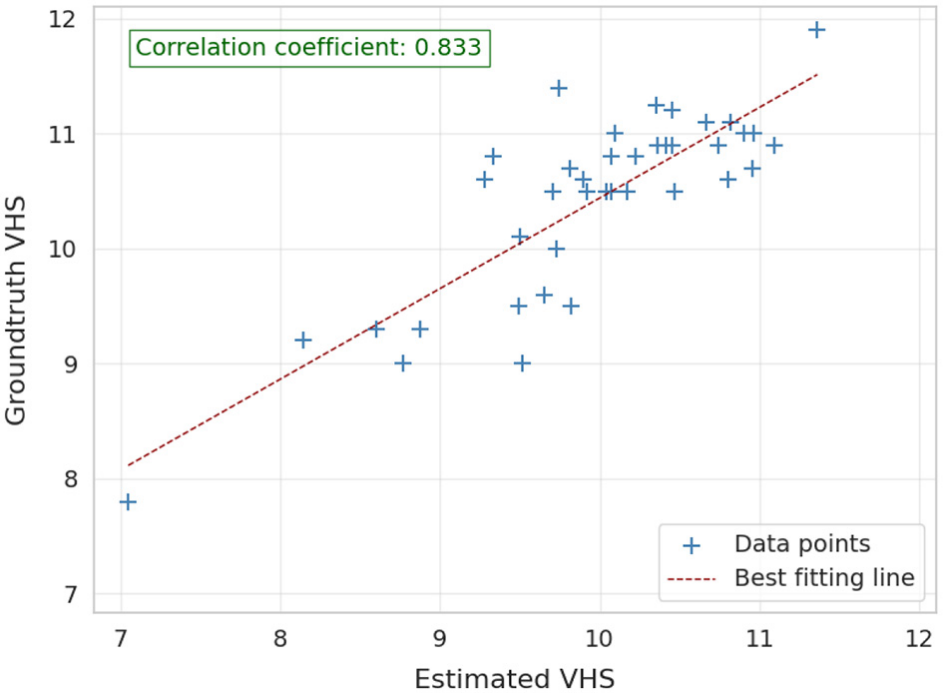
Correlation between the ground-truth and estimated VHS on validation images (40 images).

Figure 5 illustrates the cardiac axes derived from the contours of the heart and carina, demonstrating their precise orientation consistent with anatomical structure. The longest axis extends from the tracheal carina to the cardiac apex, while the short axis intersects the widest part of the cardiac silhouette perpendicularly to the longest axis, reflecting clinical practice. The VHS estimation method demonstrated significant performance, achieving a PCC of 0.833, indicating a strong linear relationship between the ground-truth and estimated values as shown in Figure 6. Additionally, the method achieved a RMSE of 0.654, reflecting its high accuracy in estimating the VHS.

### 3.2 CTR estimation model

#### 3.2.1 Performance in segmentation of the heart and thorax

**Figure 7:**
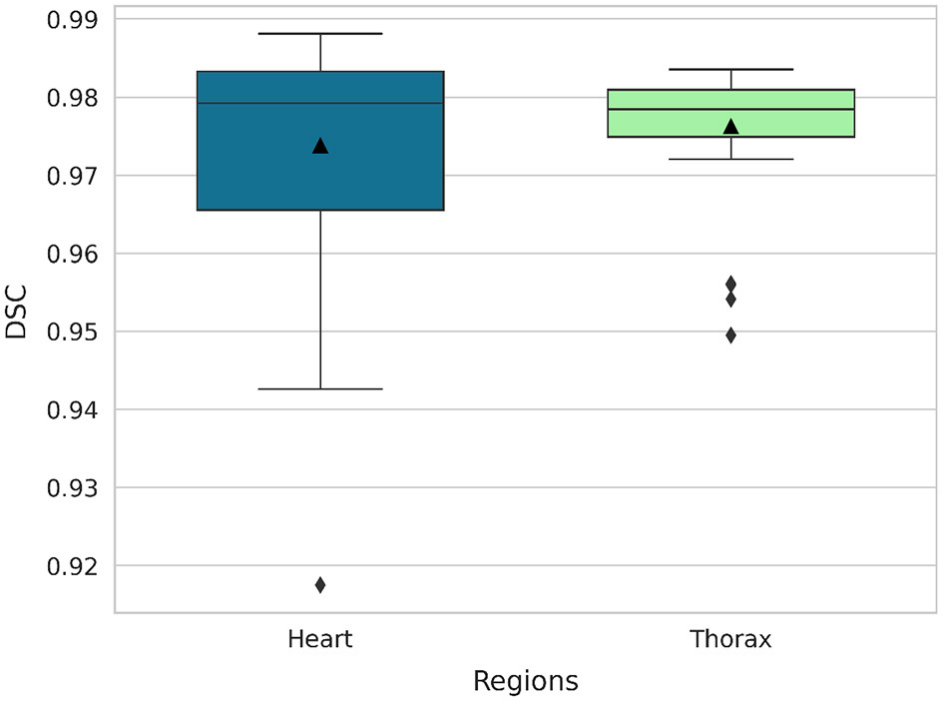
Box plots of DSC for the segmentation of the heart and thorax (40 images).

The segmentation model *M*_2_ demonstrated excellent performance in segmenting both the heart and thoracic region on the validation set, achieving an average DSC of 97.4% for the heart and 97.6% for the thorax. The median DSC values were 97.9% for the heart and 97.8% for the thorax, indicating consistently high segmentation accuracy across the dataset. Furthermore, the model maintained robust performance even in challenging cases, with minimum DSC values of 91.8% for the heart and 94.9% for the thorax. Figure 7 illustrates box plots of the DSC distributions for both anatomical structures, further highlighting the model's reliability and precision.

#### 3.2.2 Performance of CTR calculation

**Figure 8:**
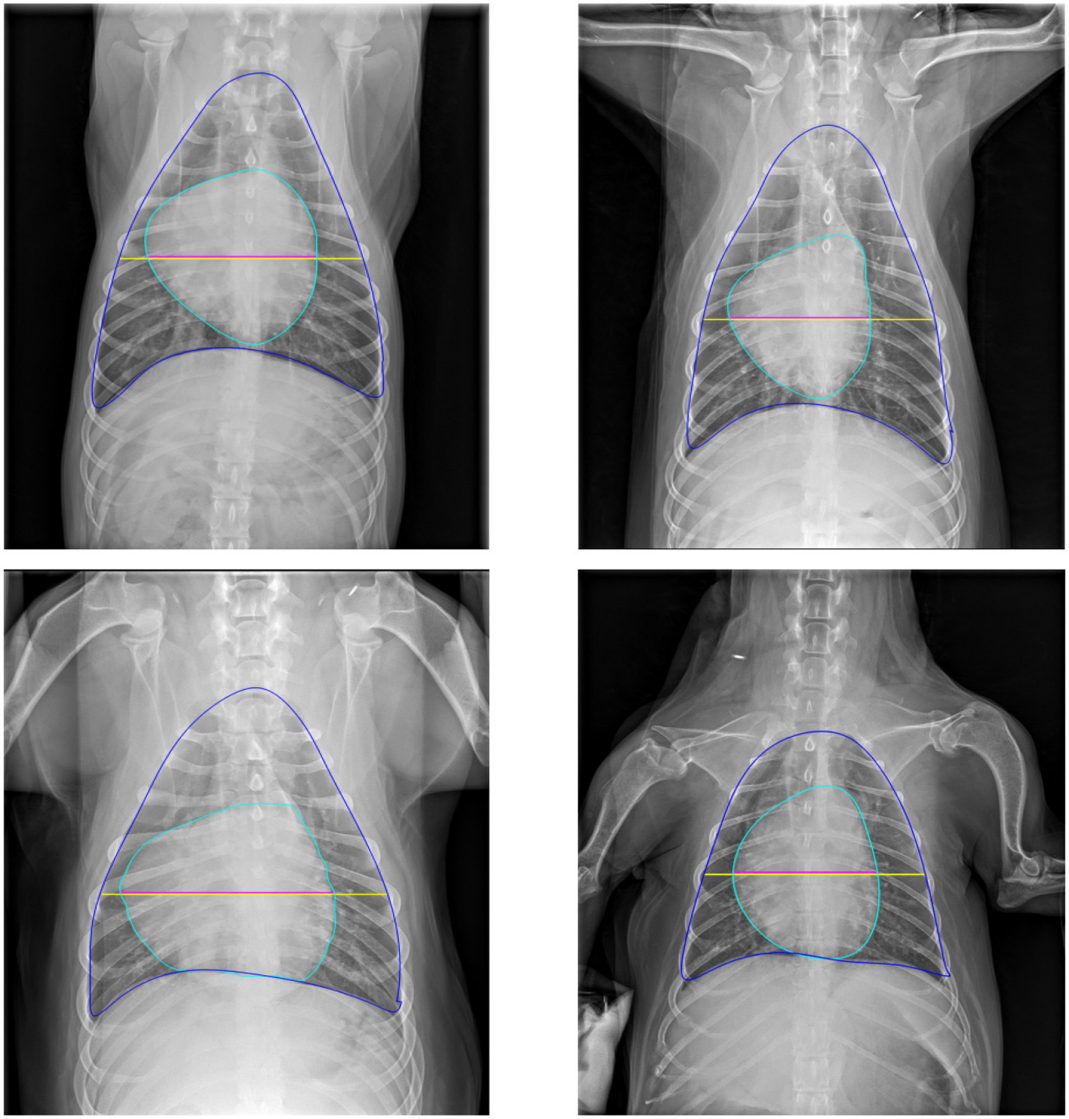
Representative examples of cardiac and thoracic size estimations on validation images. Blue and cyan contours denote the thorax and heart boundaries, respectively. The yellow line indicates thorax size, while the magenta line represents heart size.

Figure 8 provides a qualitative illustration of the model-derived measurements of heart and chest sizes, extracted from the corresponding heart and thoracic contours generated by the segmentation model. The extracted anatomical boundaries enable a visually coherent estimation of cardiac and thoracic dimensions, with results that align well with established clinical practices. While the qualitative consistency supports the model's practical applicability, the quantitative performance of CTR estimation is not presented, as ground-truth annotations for CTR were not available for the validation dataset.

### 3.3 Clinical evaluation of the deep learning algorithms

**Figure 9:**
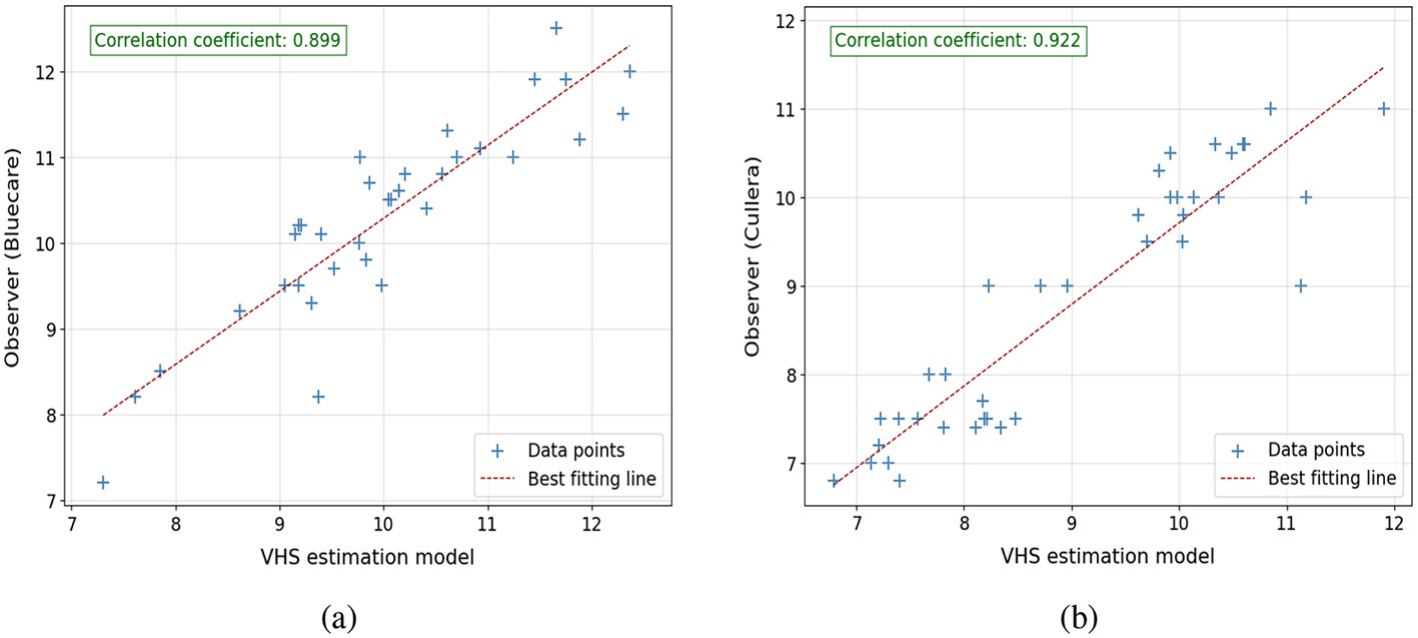
Correlation plots comparing VHS estimations from the model with observer measurements: **(a)** model estimations vs. observer from Bluecare (regression line is *y* = 0.95*x*+0.24) and **(b)** model estimations vs. observer from Cullera (regression line is *y* = 0.92*x*+0.89). Respectively, 34 and 39 cases.

**Figure 10:**
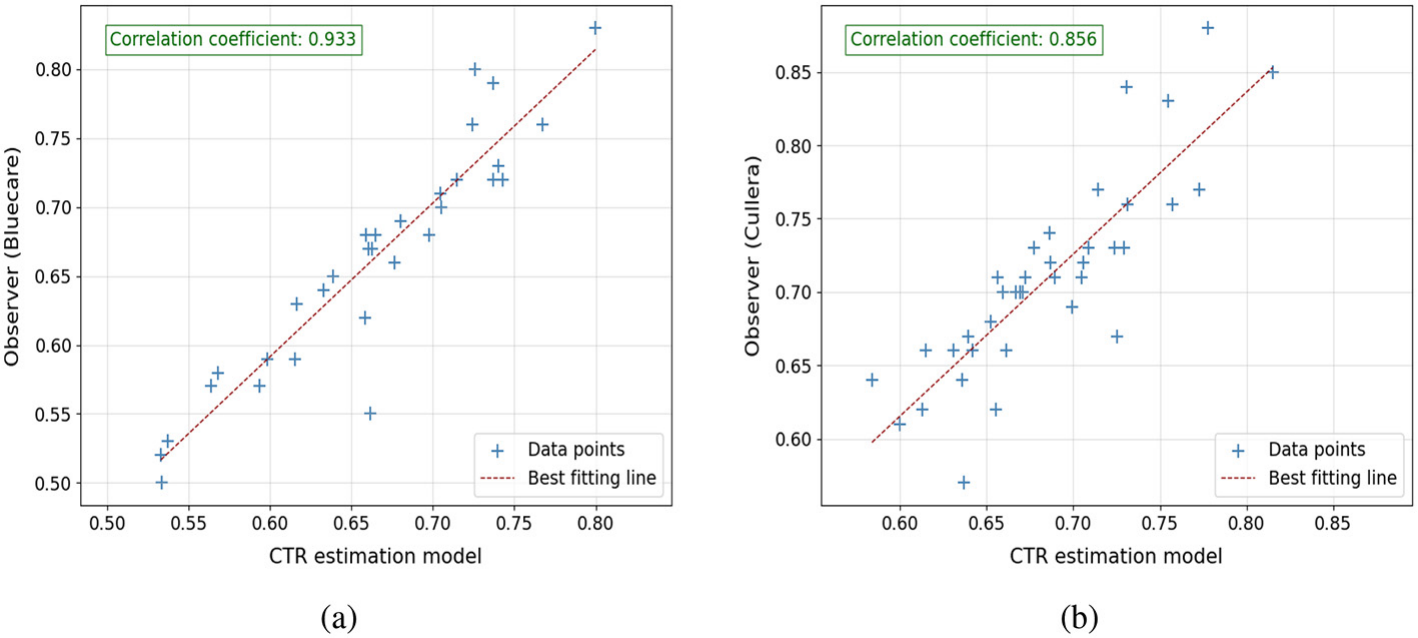
Correlation plots comparing CTR estimations from the model with observer measurements: **(a)** model estimations vs. observer from Bluecare (regression line is *y* = 0.78*x*+0.15) and **(b)** model estimations vs. observer from Cullera (regression line is *y* = 0.66*x*+0.21). Respectively, 34 and 39 cases.

The performance of the VHS and CTR estimation models was assessed relative to the manual assessments conducted by the expert teams from Cullera and Bluecare. Figures 9, 10 illustrate the correlation between automatically estimated and manually calculated VHS and CTR values, respectively, across the test dataset. We included in the caption the slope and interception of the regression fit to provide a more quantitative and interpretable measure of agreement between the automatic and manual annotations.

For VHS, the results indicate a strong linear correlation between the model predictions and the manual assessments, with PCC values of 0.899 and 0.922 for Bluecare and Cullera, respectively. The agreement between manual and automated measurements are further supported by the regression equations, yielding slopes of 0.95 and 0.92 and interceptions of 0.24 and 0.89, respectively. Notice that a slope close to 1 and a small positive intercept suggest minimal systematic bias, reinforcing the reliability and robustness of the proposed method. For CTR, we obtained strong correlations with manual measurements (PCC = 0.93 and 0.85 for Bluecare and Cullera, respectively). However, the regression analysis revealed slopes of 0.78 and 0.66 with intercepts of 0.14 and 0.21, indicating a tendency to underestimate higher values.

The root mean square error (RMSE) between the model's predictions and manual calculations further complements these findings. For VHS, the RMSE values are 0.607 and 0.587 for Bluecare and Cullera, respectively. Given that the mean VHS value is around 10 vertebral units, these deviations represent an error of approximately 6%, which indicates a high level of agreement. In terms of Mean Absolute Error (MAE), the corresponding values are 0.51 and 0.41. These results are slightly better than the RMSE of 0.654 observed on the validation dataset, showing the robustness of the algorithm. Similarly, for CTR, the RMSE values are 0.031 and 0.043, which correspond to deviations below 7% with respect to the mean CTR value of 0.66. These low errors further support the accuracy and reliability of the automatic measurements.

We can further study the performance of the algorithms computing the 95% confidence intervals for the ground-truth and the automatically estimated VHS and CTR values across the test dataset, which are shown in Table 3. For VHS, the estimated intervals closely align with the ground-truth, demonstrating the model's accuracy. In Bluecare, the estimated range slightly underestimates both the lower and upper bounds of the ground-truth, while, in Cullera, the estimated interval slightly overestimates both bounds of the ground-truth, reflecting a minimal upward shift. For CTR, the estimated intervals also show strong agreement with the ground-truth. In Bluecare, the estimated range overlaps extensively with the ground-truth, and in Cullera, the estimated interval slightly narrows variability compared to the ground-truth but remains consistent with its central tendency.

**Table 3:** Comparison of 95% confidence intervals for ground-truth and automatically estimated VHS and CTR in the observer study dataset.

**Biomarker**	**ground-truth**		**Estimated**	
	**Bluecare**	**Cullera**	**Bluecare**	**Cullera**
VHS	(9.881, 10.702)	(8.364, 9.289)	(9.577, 10.443)	(8.573, 9.497)
CTR	(0.630, 0.693)	(0.687, 0.732)	(0.637, 0.689)	(0.668, 0.703)

**Figure 11:**
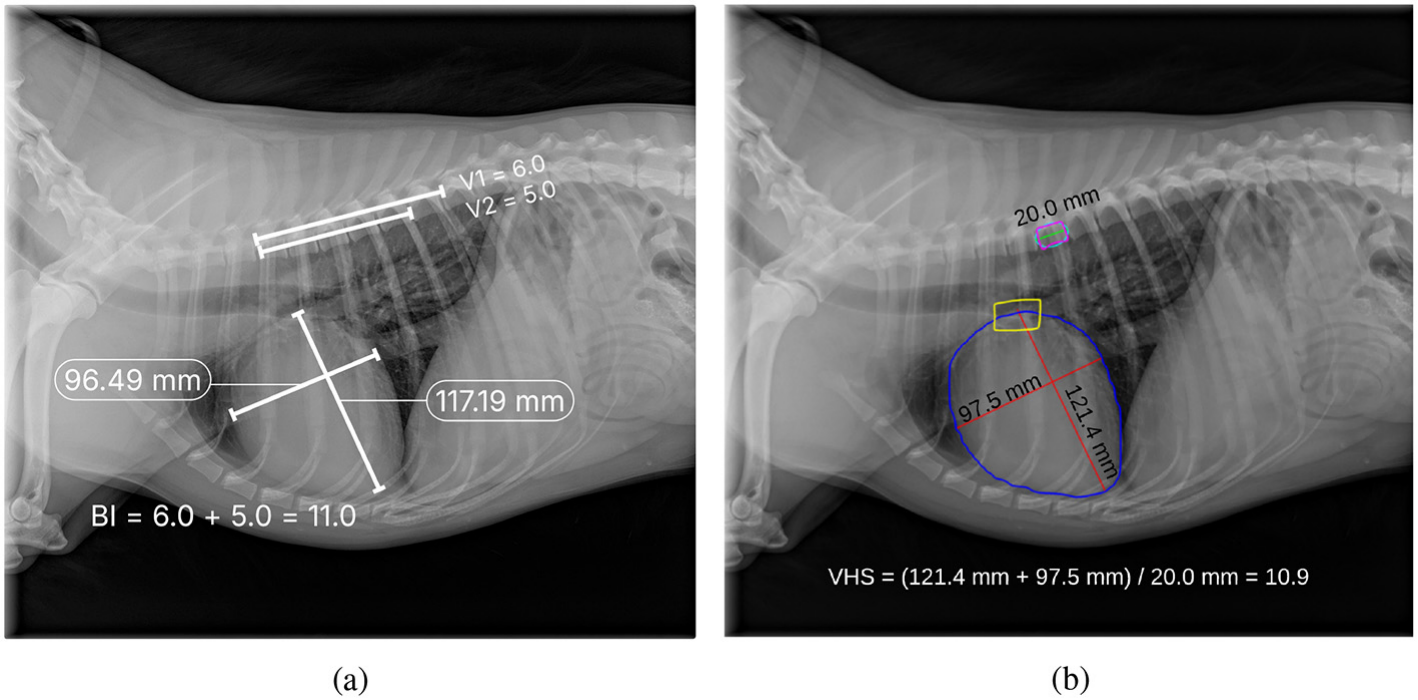
Comparison of manual annotation **(a)** and automatic estimation **(b)** of VHS, where V1 in **(a)** indicates the number of vertebrae that fall within the cardiac long axis length starting from T4, and V2 in **(a)** indicates the number of vertebrae that fall within the cardiac short axis length starting from T4.

**Figure 12:**
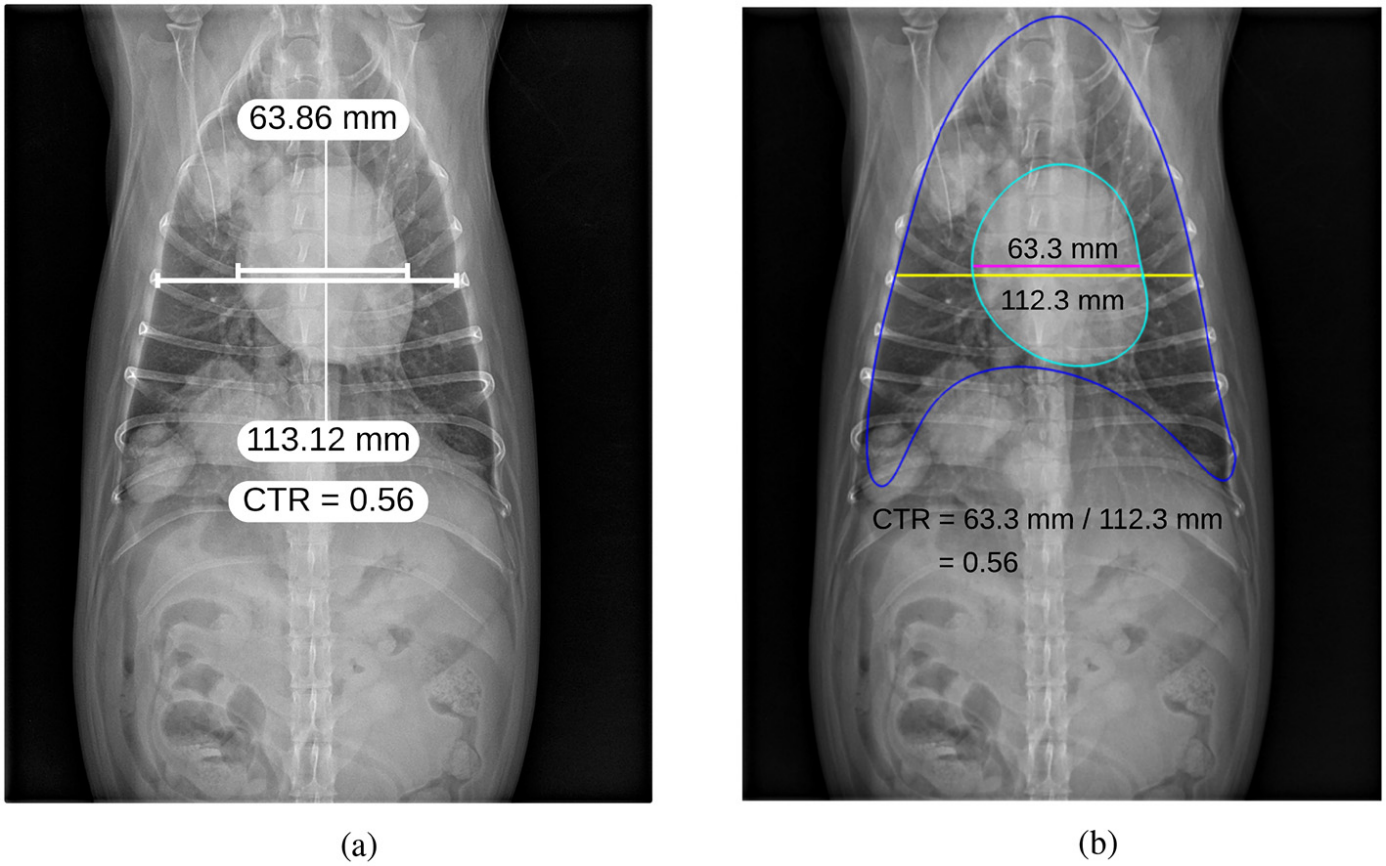
Comparison of manual annotation **(a)** and automatic estimation **(b)** of CTR.

Finally, Figure 11 presents a qualitative comparison between manual annotations and automatic VHS estimations. The figure demonstrates that the orientation of the automatically estimated cardiac axes closely aligns with the expert manual annotations. Key landmarks, such as the carina and apex, are accurately localized by the automated method, ensuring robust determination of the longest cardiac axis. Moreover, the automatically calculated VHS values are consistent with the manual measurements, underscoring the reliability of the automated system for clinical use. Similarly, Figure 12 illustrates the comparison between manual and automatic CTR estimations. The results show that the automatically estimated heart and thorax dimensions closely match the expert annotations. Additionally, the automatically calculated CTR values are nearly identical to the manual measurements, further demonstrating the accuracy and effectiveness of the automated approach in providing precise clinical assessments.

### 3.4 Human observer study

**Figure 13:**
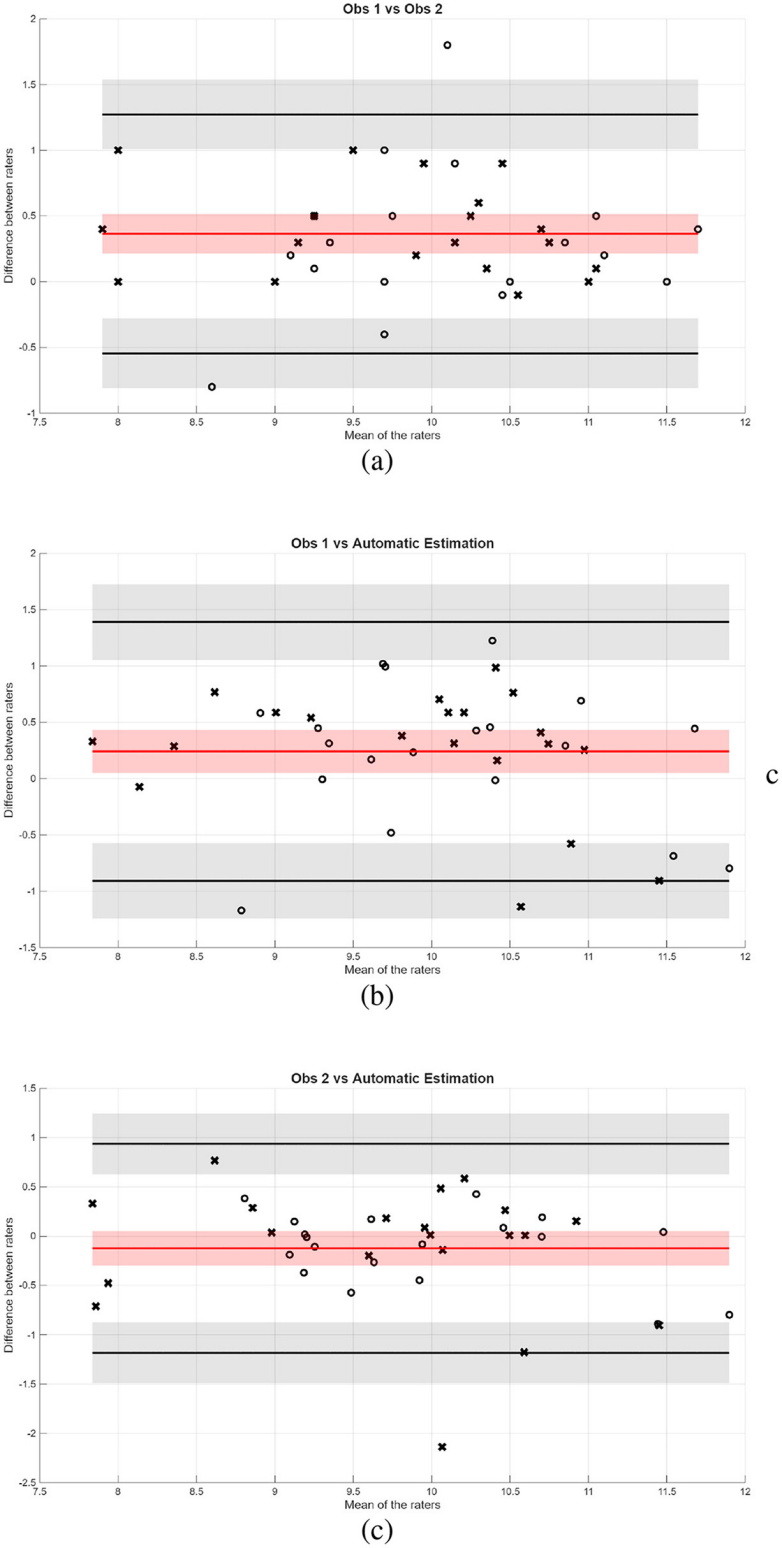
Bland-Altman plots between **(a)** raters from the two centers, **(b)** rater from Bluecare vs. automatic estimation, and **(c)** rater from Cullera vs automatic estimation, when estimating VHS from LM images.

To compare the performance of the automatic tools with human raters, we performed an inter-observer study with a subset of the images. In this study, images from both centers were independently evaluated by the experts' teams of both centers. The results for VHS estimation are shown in terms of Bland-Altman plots in Figure 13. In the plots, circles and crosses were used to denote the acquisition center of each image.

The Bland-Altman plot comparing Observer 1 and Observer 2 (the two clinical centers) shows a mean bias of 0.36, indicating a systematic difference between the two raters. However, given that the VHS measurement range lies between 8 and 12, a bias of 0.36 remains relatively small (3%−4%) and may be considered acceptable on the required level of precision for clinical interpretation.

On the other hand, the comparisons between manual observations and the automatic estimation method reveal lower biases: 0.2412 for Observer 1 and −0.1229 for Observer 2 (2%−3% and 1%−1.5%, respectively). These values suggest a better systematic agreement with the automatic method than between the two manual raters. Across all three comparisons, the 95% limits of agreement were of similar magnitude, indicating comparable variability. Most points lay within these limits, with only a few outliers observed. In general, the Bland-Altman analysis indicates that the automatic method could be a viable alternative.

**Figure 14:**
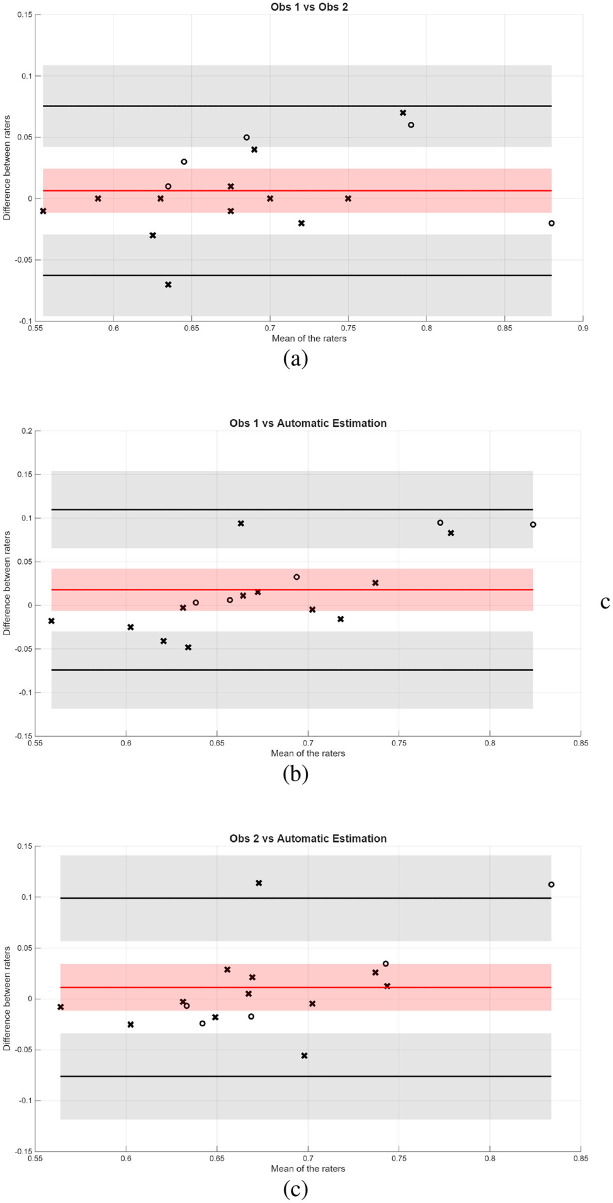
Bland-Altman plots between **(a)** raters from the two centers, **(b)** rater from Bluecare vs. automatic estimation, and **(c)** rater from Cullera vs. automatic estimation, when estimating CTR from VD images.

Figure 14 shows the Bland-Altman plots for the CTR measurement. In this case, the Bland-Altman plot comparing the two clinical centers shows more agreement than in the VHS case, with a bias of 0.065 (only 1% of disagreement). In contrast, the comparisons of each observer with the automatic estimation display show larger mean biases (0.0178 and 0.0113, respectively) and slightly wider limits of agreement, indicating greater variability between human and automated measurements. Overall, the automated approach broadly agrees with human ratings but is less precise than inter-observer agreement.

## 4 Discussion

This study aimed to develop a deep learning framework for automatic estimation of VHS and CTR in dogs and cats from thoracic radiographs, leveraging Mask R-CNN-based segmentation models and subsequent anatomical measurements. The results obtained underscore the robustness of the proposed methodology, with strong correlations between automated predictions and manual assessments, while also identifying notable areas for improvement.

The T6 vertebra was used to normalize the patient's heart size relative to body size in this study. The use of the T4 vertebra as a normalizing factor, as described in Jeong and Sung (8), was also investigated. However, the experiments demonstrated a stronger correlation with the T6 vertebra. This may be attributed to the T6 vertebra's central position within the thoracic spine, which likely provides a more representative and consistent reference point for normalizing heart size across diverse patients. Future research could explore the inclusion of intervertebral space measurements to further refine VHS estimation.

The segmentation model *M*_1_ demonstrated significant performance in segmenting the heart, achieving a mean DSC of 94.2%. However, its performance was comparatively lower for more challenging structures, such as the T6 vertebra and the carina, which yielded mean DSCs of 62.6% and 68.9%, respectively. The segmentation inaccuracies for the T6 vertebra primarily stemmed from structural similarities and relative positional variations among adjacent vertebrae (T5, T6, and T7). Despite these issues, it is noteworthy that the VHS calculation process remained robust to these variations, as adjacent vertebrae exhibit similar dimensions. When excluding cases of mis-segmented vertebrae, the mean DSC for the T6 vertebra improved significantly to 80.8%, emphasizing the potential for enhanced performance with refined training data or additional anatomical landmarks for differentiation. Similarly, the carina, being a relatively small and anatomically complex structure, posed a challenge for accurate segmentation due to its indistinct and variable boundaries across different radiographs.

The segmentation model *M*_2_ showcased outstanding performance in segmenting the heart and thoracic regions, achieving mean DSCs of 97.4% and 97.6%, respectively. These results highlight the model's ability to generalize effectively across a diverse testing dataset. This high segmentation accuracy significantly contributed to the reliable calculation of the CTR, demonstrating the model's potential for seamless integration into clinical workflows.

The VHS estimation model exhibited strong agreement with manual assessments, achieving a PCC of 0.833 and an RMSE of 0.654 on the testing dataset. These metrics show the model's ability to provide accurate and consistent measurements aligned with radiological practices. Additionally, the method's robustness was highlighted in observer study datasets, where PCCs of 0.899 (Bluecare) and 0.922 (Cullera) were achieved with slope similar to 1 and intersection close to 0, suggesting no bias in the results. These results demonstrate the model's effectiveness in adapting to real-world variability in radiographs. In addition, the CTR estimation also achieved a comparable level of agreement with manual measurements, reflected in a PCC of 0.933 (Bluecare) and 0.856 (Cullera), with RMSE values of 0.031 for Bluecare and 0.043 for Cullera. The consistency in CTR measurements can be attributed to the high accuracy of *M*_2_ in segmenting the thoracic and cardiac regions, highlighting the reliability of the framework for detecting cardiomegaly in ventrodorsal radiographs.

The clinical study, performed using the test dataset, demonstrated strong correlations between automated and manual assessments, validating the clinical applicability of the proposed framework. The mean absolute error between automatic and manual VHS measurements was approximately 0.5 cm. This is comparable to the mean intra-group difference of 0.5 vertebral units (*v*) reported by Hansson et al. (18) for human observers, and significantly lower than the interobserver difference of 1.0 ± 0.3*v*. Such a level of agreement suggests that our automated method performs on par with trained human experts. Similarly, we observed differences between the Bluecare and Cullera observers, which may reflect interobserver variability and potentially image acquisition differences. These findings underscore the value of automated tools in standardizing measurements, reducing subjectivity, and improving consistency across clinical settings.

Direct comparisons with state of the art are challenging due to differences in datasets and methodologies. Some state-of-the-art approaches focus on image classification without providing quantitative measurements, while others rely on anatomical landmark detection, which is not fully aligned with clinical procedures. In contrast, we conducted an inter-observer study comparing automated predictions with annotations from two centers. Bland-Altman plots show that automated VHS predictions were comparable to, or even exceeded, the agreement observed between manual raters. On the other hand, CTR estimation showed slightly larger discrepancies than those between human raters, possibly because this task is relatively straightforward for experts.

A key limitation of this study is the relatively small size of the training dataset, which reduced the robustness and generalizability of the segmentation model *M*_1_, particularly when dealing with anatomically variable or less distinguishable structures. This constraint is common in veterinary imaging, where curated and annotated datasets are typically limited in size due to the diversity of species, breeds, and imaging protocols, as well as the lack of large-scale public repositories. Although we mitigated this limitation by initializing the segmentation model with weights pre-trained on the COCO dataset (transfer learning), which provided a strong starting point for learning general visual features, some anatomical regions remain challenging. Addressing this issue could involve expanding the dataset to include more diverse and representative cases, especially those with complex or atypical anatomical presentations, thereby enhancing the model's ability to capture broader anatomical variability. In addition, exploring data-centric strategies such as synthetic image generation or semi-supervised learning methods could further improve model performance by leveraging unlabeled data or augmenting training diversity without requiring extensive manual annotation.

The proposed framework offers significant potential for veterinary medicine, particularly in scenarios where experienced radiologists are scarce. By providing automated, accurate, and reproducible measurements of VHS and CTR, the framework can assist veterinarians in the early detection and monitoring of cardiac conditions, potentially improving patient outcomes. The strong agreement of automated predictions with radiologists' assessments highlights its potential for future integration into clinical workflows.

## 5 Conclusion

In conclusion, this study highlights the effectiveness of deep learning-based approaches for the automated estimation of key cardiac biomarkers in veterinary radiology. While further refinements are necessary to address segmentation challenges, the demonstrated accuracy and reliability of the framework underscore its potential as a valuable tool in veterinary practice.

Future efforts will aim to overcome segmentation challenges by expanding the training dataset and leveraging advanced deep learning techniques, such as attention mechanisms or transformer-based models. Further validation on larger and more diverse datasets will be crucial for enhancing the framework's generalizability and clinical applicability. Ultimately, developing an end-to-end tool with real-time prediction capabilities could facilitate its adoption in veterinary practices, improving early detection and management of cardiac conditions in pets.

## Data Availability

The data analyzed in this study is subject to the following licenses/restrictions: The data can be made available upon request to the corresponding author. Requests to access these datasets should be directed to Robert Marti, robert.marti@udg.edu.

## Footnotes

^1^ https://docs.ultralytics.com/

^2^ https://labelme.io/
